# Recent Advances in Molecularly Imprinted Polymers and Emerging Polymeric Technologies for Hazardous Compounds

**DOI:** 10.3390/polym17081092

**Published:** 2025-04-18

**Authors:** Ana-Mihaela Gavrilă, Mariana Ioniță, Gabriela Toader

**Affiliations:** 1 National Institute for Research, Development in Chemistry and Petrochemistry ICECHIM, 202 Spl. Independentei, 060021 Bucharest, Romania; ana.gavrila@icechim.ro; 2 Advanced Polymer Materials Group, National University of Science and Technology POLITEHNICA Bucharest, Gheorghe Polizu 1-7, 011061 Bucharest, Romania; mariana.ionita@polimi.it; 3 Military Technical Academy “Ferdinand I”, 39-49 George Cosbuc Boulevard, 050141 Bucharest, Romania

**Keywords:** molecularly imprinted polymers, polymeric platforms, isolation, sensing, decontamination, hazardous compounds

## Abstract

Addressing hazards from dangerous pollutants requires specialized techniques and risk-control strategies, including detection, neutralization and disposal of contaminants. Smart polymers, designed for specific contaminants, provide powerful solutions for hazardous compound challenges. Their remarkable performance capabilities and potential applications present exciting opportunities for further exploration and development in this field. This editorial aims to provide a comprehensive overview of smart materials with unique features and emerging polymeric technologies that are being developed for isolation, screening, removal, and decontamination of hazardous compounds (e.g., heavy metals, pharmaceutically active contaminants, hormones, endocrine-disrupting chemicals, pathogens, and energetic materials). It highlights recent advancements in synthesis methods, characterization, and the applications of molecularly imprinted polymers (MIPs), along with alternative smart polymeric platforms including hydrogels, ion-imprinted composites, screen-printed electrodes, nanoparticles, and nanofibers. MIPs offer highly selective recognition properties, reusability, long-term stability, and low production costs. Various MIP types, including particles and films, are used in applications like sensing/diagnostic devices for hazardous chemicals, biochemicals, pharmaceuticals, and environmental safety.

In contemporary society, the issues of environmental health and safety have become increasingly significant, reflecting the growing awareness of the challenges posed by hazardous materials. This reality underscores the need for advanced technologies and the establishment of new approaches to manage and mitigate these threats effectively. Such initiatives are vital for protecting both ecosystems and human health, promoting a holistic approach to safeguarding environmental and public health.

Addressing hazards from dangerous pollutants in water, air, food, and land resources requires specialized techniques and risk-control strategies, including detection, identification, neutralization, removal, and disposal of contaminants. Gavrila and Toader described a newsworthy overview comprising recent studies targeting hazardous substances, including drugs, explosives, chemical warfare agents, pollutants, and biological or chemical agents, along with their detection and decontamination methods using smart polymeric platforms [1].

In this context, versatile, advanced polymeric materials have attracted considerable interest due to their unique responses to external factors. Their remarkable performance capabilities and potential applications present exciting opportunities for further exploration and development in this field. Smart polymers, or stimuli-responsive polymers, react to various stimuli such as pH changes, temperature, solvents, and biological or chemical agents, leading to changes in their structural, chemical, mechanical, optical, or electrical properties [2].

Another powerful solution to address hazardous compound challenges is the significant progress in developing molecularly imprinted polymers (MIPs), one of the most available and tailor-made biomimetic receptors with similarities to antibodies [3]. MIPs offer highly selective recognition properties, reusability, long-term stability, and low production costs. Various MIP types, including particles, nanofibers, and films, are used for a wide range of applications, such as sensing/diagnostic devices for hazardous chemicals, biochemicals, pharmaceuticals, environmental safety or decontamination purposes, and extraction/purification protocols of active ingredients.

In response to these inquiries, this editorial aimed to provide a comprehensive summary of emerging polymeric technologies that are being developed for isolation, screening, removal, and decontamination of hazardous-type compounds and derivatives, as well as mitigation solutions. We spotlight recent advancements not only in current synthesis methods and characterization but also regarding the real tools using alternative polymeric platforms orchestrated by MIPs, hydrogels, ion-imprinted composites, screen-printed electrodes, nanoparticles, and nanofibers. Herein, the proposed emerging polymeric technologies or techniques cover molecular or ion imprinting, electrochemical sensing such as voltammetry, “eco-friendly” coating technology, wastewater treatments such as adsorption-based strategies, electrospinning process, and inactivation procedures.

Water pollution caused by ecotoxicants is a pressing environmental challenge, and among these pollutants, metals present a particularly grave threat to human health. The presence of toxic substances in our water systems poses a serious threat not only to ecosystems but also to our overall well-being. When these substances exceed permissible concentration levels, they become hazardous, prompting many countries to establish stringent regulations regarding metal ions in water. Lead (Pb), for instance, is a common metallic pollutant that poses significant risks to both environmental systems and human health. In a study conducted by Yixin Sui et al. [4], a composite material was developed, imprinted with Pb (II), utilizing modified sand particles as carriers. It is relevant to mention here an advanced technology that specializes in the recognition and adsorption of specific ions, namely ion-imprinted polymers (IIPs), a kind of extension of MIPs. This material exhibits remarkable selectivity and efficiency in removing Pb (II) from aqueous solutions, showcasing its potential for environmental remediation. Even in the presence of interfering ions, it retains strong selectivity for Pb (II) and demonstrates an adsorption capacity of 41.83 mg·g^−^^1^. Another study, by Alma Khassenovna Zhakina et al. [5], investigated the development and properties of a zinc-imprinted polymer (ZnIP) made from humic acids obtained from Shubarkol coal. The ZnIP effectively captures zinc ions, positioning it as a valuable solution for treating zinc-contaminated water. The research demonstrates that the ZnIP can selectively extract zinc ions from complex mixtures, including wastewater, emphasizing its potential to address both environmental and technological challenges.

Among the various protocols for wastewater decontamination, methods that employ adsorbent materials are often regarded as particularly advantageous. In addition to MIPs, various polymeric platforms, such as hydrogels, offer effective solutions for the removal of heavy metals [6]. These materials also exhibit considerable potential in addressing environmental challenges associated with metal/heavy metal contamination. In a recent overview by Persano et al. [7] on the removal of metal ions/heavy metals from water using bio-inspired hydrogels, the possibility of developing new hydrogels with improved sorption performances was evidenced. Their cost-effectiveness, simplicity, efficiency, and scalability make them suitable choices for treating substantial volumes of contaminated water. The aforementioned work focuses on the main developments regarding the preparation techniques of cellulose-based hydrogels as smart polymers and their derivatives. The findings regarding the adsorption capacity for different heavy metal ions are between 2.3 and 2240 mg·g^−^^1^.

Moreover, pharmaceutical pollution poses a worldwide threat to environmental and human health; thus, effective removal of other types of pharmaceutically active contaminants [8] in various waters, soils, and activated sludges are of extensive research, such as antibiotics, analgesics, and hormones. Other pharmaceuticals include persistent pollutants antiretroviral drugs (ARVs), antidepressants/antiepileptics, and non-steroidal anti-inflammatory drugs (NSAIDs) [9]. To date, various processes for decontamination of emerging pharmaceutical pollutants have been developed, e.g., advanced oxidation and coagulation–flocculation processes, safer methods such as biodegradation and nano-filtration or using environmentally friendly nanomaterials, biocatalysts, and MIPs. Sisonke Sigonya et al. [10] provide valuable insights into the synthesis and characterization of electrospun MIP composite membranes composed of polyvinyl alcohol (PVA) and polyethylene terephthalate (PET). The research underscores the ability of electrospun fiber technology to improve selective adsorption through customized MIP designs, which provide specific binding sites for targeted pharmaceutical pollutants, such as ARVs and NSAIDs. It investigates the electrospinning and rheological characteristics of PET and PVA with different levels of hydrolysis for MIP integration, showing that PVA nanofibers demonstrate favorable attributes for use in filtration and sensing applications. The resulting imprinted composite membranes revealed high selectivity and excellent adsorption performances, leading to a mitigation solution regarding the environmental impact of emtricitabine and tenofovir disoproxil as drugs and further addressing the public health concerns associated with their presence in various water supplies. As already mentioned, hormones are harmful compounds that can be removed and detected from wastewater from domestic actions, including endocrine-disrupting chemicals (EDCs), such as estradiol, a phenolic steroid estrogen. In this context, Koç et al. [11], prepared a new generation of polymeric composite, 17β-estradiol-imprinted (Hydroxyethyl)methacrylate-based nanoparticles onto bacterial cellulose nanofibers. The resulting adsorbents based on the composite nanofibers demonstrated a high fast binding capacity of 160.05 µg·g^−^^1^ and a high selectivity upon cholesterol and stigmasterol as competing steroids. This study demonstrates that composite imprinted nanofibers offer an important, long-lasting approach for removing EDCs.

Likewise, prolonged exposure to wastes and sewage treatment facilities is linked to the presence of antibiotic-resistant bacteria. Alongside EDCs, pathogens considered hazardous CBRN contaminants, including *S. aureus* and *E. coli*, can be detected and/or isolated using advanced polymeric innovative platforms [12]. Toader et al. prepared nanocomposite hydrogel films based on sequential interpenetrating polymeric networks with remarkable mechanical performances as drug-delivery platforms [13]. The method is based on the synergetic features brought by the combination of two organic polymer matrices (poly (N-vinylpyrrolidone) and sodium alginate), interconnected by both covalent and ionic crosslinking, with multifunctional nanosized fillers (bentonite, TiO_2_, ZnO) with a demonstrated capacity to act as a delivery platform for nafcillin. The authors demonstrated that the nafcillin loaded into the nanocomposite hydrogel films ensured high to moderate activity against *S. aureus* and *S. pyogenes* and no activity against *E. coli*, as hazardous-type compounds.

The potential of MIPs to enhance the isolation and purification of specific bioactive compounds was revised by highlighting the use of molecular imprinting technology for extracting and detecting Quercetin in plants [14]. This article reviews the advancements in molecular imprinting technology used for the extraction and detection of Quercetin, a flavonoid compound known for its antioxidative, anti-aging, and anti-cancer properties. The study emphasizes the high selectivity and sensitivity of MIPs in isolating and analyzing Quercetin from complex plant matrices. Bioactive compounds, such as Quercetin, can mitigate hazardous materials (HAZMATs) by neutralizing toxic substances. These compounds have shown potential in controlling food-borne viruses and contaminants [15], thus reducing the impact of hazardous compounds on human health and the environment. Integrating bioactive compounds into MIP-based systems can lead to more effective and eco-friendly solutions for managing HAZMAT risks.

As a new screening method for biomedical tests, modified electrodes for on-site or point-of-care detection [16] for various hazardous-type compounds are in high demand [17]. Far apart from traditional electrodes, screen-printed electrodes (SPEs) are popular thanks to their tunable custom-designed properties (e.g., flexibility and portability for onsite applications, ease of use and production) through embedding and/or immobilizing nano-structured materials [18]. Daniele Merli et al. [19] introduced a quantitative method for dopamine determination using a molecularly imprinted polypyrrole (e-MIP)-modified screen-printed electrode and differential pulse voltammetry (DPV). While dopamine itself is not intrinsically harmful, its dysregulation can result in significant health concerns. The e-MIP-SPEs exhibited a detection limit (LOD) of 0.8 µM, remarkable selectivity, and sensitivity of 0.078 µA µM^−1^, establishing it as a reliable tool for monitoring dopamine levels in both clinical and research contexts. Hence, the interference tests and practical application are performed using analysis of synthetic and human urine samples, with recoveries ranging from 87% to 103% for all samples. By utilizing the insights from this study, researchers can investigate the application of MIPs and advanced analytical methods for detecting hazardous materials.

Despite the ongoing threats posed by chemical, biological, radiological, and nuclear (CBRN) hazards, the use of energetic materials (EMs) continues to pose severe environmental and human risks due to their hazardous nature and toxicological properties. A heightened priority has been directed towards ‘greener’ approaches and EMs, including formulations of polymer-bonded explosives (PBXs), which are essential for enhancing global security measures and mitigating the environmental impact of military operations. Among EMs, nitramines such as hexogen (RDX) and octogen (HMX) have been found in the soil and water from many government military bases due to improper storage, weapons testing, and production. The development of ‘green’ PBX formulations minimizes the risks associated with hazardous materials, providing a more environmentally responsible solution. Rotariu et al. introduced an innovative method for producing “green” PBX formulations utilizing two high explosives, RDX and HMX, combined with acrylic acid–ethyl acrylate copolymeric binders [20]. This approach showcases an eco-friendly technology for coating RDX and HMX crystals, leveraging the adjustable water solubility of the polymeric binders at a basic pH. By doing so, it eliminates the need for traditional organic solvents and facilitates a safer and more straightforward PBX manufacturing process. The resulting formulations exhibited high energetic performance, alongside the ability to easily recover the explosive crystals through the dissolution of the polymeric binder at pH 11 and 30 °C.

The publications discussed contribute positively to the development and application of advanced polymeric materials, providing valuable solutions to address important environmental and health challenges. Collectively, these studies highlight the versatility and effectiveness of imprinting techniques and other advanced polymeric technologies in mitigating the impact of various hazardous compounds, including heavy metals, pharmaceutically active contaminants, hormones, EDCs, pathogens, and EMs. The articles focus on the synthesis and characterization of novel materials that exhibit high selectivity, sensitivity, and stability. These materials demonstrate significant potential in areas such as environmental remediation, pollution control, and drug delivery. Several studies also explore eco-friendly and sustainable approaches, including “green” PBX and cellulose-based hydrogels, highlighting the importance of developing environmentally responsible solutions. Overall, the common theme among these articles is the innovative application of polymer technologies to detect, remove, and neutralize hazardous substances, which contributes to the advancement of environmental safety and public health.
